# Parkinson's Disease: New Insights into Pathophysiology and Rehabilitative Approaches

**DOI:** 10.1155/2016/3121727

**Published:** 2016-06-30

**Authors:** Vincenza Frisardi, Andrea Santamato, Binith Cheeran

**Affiliations:** ^1^Geriatric and Rehabilitation Department, Golgi Redaelli Institute, Abbiategrasso, 26180 Milan, Italy; ^2^Medical & Surgical Emergency Department, “Carlo Poma” Hospital, 46100 Mantua, Italy; ^3^Department of Physical Medicine and Rehabilitation, University of Foggia, 71100 Foggia, Italy; ^4^Nuffield Department of Clinical Neurosciences, John Radcliffe Hospital, Level 6, West Wing, Oxford OX3 9DU, UK

Parkinson's disease (PD) is a multifactorial neurodegenerative disorder, but up till now, its etiology remains largely unknown. Progressive impairment of voluntary motor control, which represents the primary clinical feature of the disease, is caused by a loss of midbrain substantia nigra dopamine (DA) neurons. PD prevalence increases steadily with age. Therefore, with the aging population, we will see an increase in social and health impact of this disease. Currently, the drugs available improve physical performances and lengthen life expectancy and autonomy in the activities of daily living by reducing adverse events; it follows that they improve the quality of life of patients. However, these drugs are symptomatic and not curative. When the patient reaches the maximum allowable therapeutic dosage of levodopa, we have to face great difficulties and impotence in disease's management.

Recently, the role of rehabilitation in the field of neurodegenerative diseases is becoming more and more important as it results from several studies. In this special issue, we wanted to focus our attention on the “holistic view” of the disease because we believe that only a multidisciplinary approach could help to face it in the best way. PD involves miscellaneous systems (physical, mental, and behavioral structures) affecting also the autonomy in self-care management. Furthermore, in this special issue we wanted to draw the attention to the importance of rehabilitation in PD.

An amount of epidemiological evidence stressed the importance of the relationships between genetic and environmental factors. L. Polito et al. summarize evidence on gene-environment interplay in the development of PD focusing on susceptibility factors and causal genetic mutation. To the best of our knowledge, this review is the first attempt to analyze all those genetic factors that modify the impact of environmental exposure.

Brilliantly, F. Magrinelli et al. contributed to examining the neuropathology, neuropharmacology, and neurophysiology of motor dysfunction of PD, with particular attention to those clinical features that impair quality of life and functional ability of patients. Starting from anatomic basis of the main circuit involved in PD, subsequently they analyzed the pathophysiology of PD motor symptoms (bradykinesia and tremor rigidity) and signs (postural balance, gait impairment, fatigue, and pain).

Currently, pharmacological treatment of motor symptoms of PD depends mainly on the administration of dopaminergic drugs (levodopa and dopamine agonists) and other drugs involved in levodopa metabolism (monoamine oxidase-B inhibitors and catechol-O-methyltransferase inhibitors). Usually, in the late disease stages, PD patients treated by dopaminergic drugs display various complications, such as* on-off* fluctuations of Parkinsonian symptoms and dyskinesia. Unfortunately, most patients with advanced PD are unsatisfactorily controlled by the usual therapeutic approach. A. Daniele et al. examine other pharmacological targets regardless of the dopaminergic pathway. In particular, they focus on potential beneficial effects of zolpidem, a positive allosteric modulator of GABAA receptors, on the motor symptoms of PD. In fact, some studies have previously suggested that there is a high density of zolpidem binding sites in the globus pallidus and in the substantia nigra, which are the output structures of the basal ganglia.

Two experimental works complete this brief special issue. The first is a case control study carried out by M. Tramontano et al. with the aim of investigating the efficacy of a blindfolded balance training in the improvement of gait parameters in people with PD compared to patients who underwent a standard physical therapy program.

The second is a prospective open-label feasibility study evaluating the impact of 8-week action observation training (video-therapy) for the treatment of postural instability and balance impairment in PD patients. Although in this latter study A. Santamato et al. did not find positive results, they underline one of the most important shortcomings in PD rehabilitation field. In fact, absence of standard procedures to improve balance and posture in PD subjects makes this area a virgin field to explore.

We hope that the contents of this special issue may help clinicians better understand the rationale of current pharmacological and rehabilitation strategies for PD and give viability to further studies that integrate current knowledge with innovations in the field of rehabilitation.

*Vincenza Frisardi* *Vincenza Frisardi* *Andrea Santamato* *Andrea Santamato* *Binith Cheeran* *Binith Cheeran*

